# The role of social relationships in the link between olfactory dysfunction and mortality

**DOI:** 10.1371/journal.pone.0196708

**Published:** 2018-05-16

**Authors:** Carrianne J. Leschak, Naomi I. Eisenberger

**Affiliations:** Department of Psychology, University of California Los Angeles, Los Angeles, California, United States of America; Instituto Cajal-CSIC, SPAIN

## Abstract

Recent work suggests that olfactory dysfunction is a strong predictor of five-year mortality in older adults. Based on past work showing: 1) that olfactory dysfunction impairs social functioning and 2) that social ties are linked with mortality, the current work explored whether impairments in social life mediated the relationship between olfactory dysfunction and mortality. Additionally, based on work showing gender differences in the social consequences of olfactory dysfunction, gender was assessed as a potential moderator of this association. Social network size mediated the olfactory-mortality link for females. To probe what feature of social networks was driving this effect, we investigated two subcomponents of social life: emotional closeness (e.g., perceived social support, loneliness) and physical closeness (e.g., physical contact, in-person socializing with others). Physical closeness significantly mediated the olfactory-mortality link for females, even after controlling for social network size. Emotional closeness did not mediate this link. Possible mechanisms underlying this relationship are discussed.

## Introduction

Human olfaction has been understudied due to the misconception that olfaction is not as important in humans relative to other animals [1]. However, recent work has demonstrated a strong link between olfactory dysfunction and increased mortality in a sample of American older adults [2]. One mechanism through which olfactory decline may predict mortality is by negatively impacting interpersonal functioning. Specifically, since olfaction is critical for normative social behavior [3,4] and social relationships are critical for health and reduced mortality (see [5] for review), olfactory dysfunction may impair interpersonal functioning, thereby increasing mortality risk.

Animal models have highlighted the importance of olfaction for social behavior in animals [3,6,7,8]. For instance, olfactory cues are important for the recognition of conspecifics [7], and olfactory dysfunction in animals can lead to unusual behavioral responses to conspecifics in different situations (e.g. nonaggressive behavior towards foreign conspecifics [3]). Most animal work, however, has focused on the effect of olfactory bulb lesions on sexual and dominance behavior in males. Considerably less work has looked at the effects of olfactory dysfunction on social behaviors that might be critical for maintaining social bonds. However, researchers have suggested that olfactory dysfunction in hamsters seems to lead to a general “social agnosia” [9], often leading to a general disinterest in conspecifics (e.g. elimination of mating behavior [10] and of territorial aggression [11]. Further, two studies have shown, that for the highly social prairie vole, olfactory dysfunction leads to less time spent with familiar others and more time spent alone [4,12]. This past work suggests that olfactory dysfunction may lead to active distancing and isolation from others.

Not surprisingly, there has been little research on the social implications of olfaction in humans, in part because there has instead been a focus on other sensory systems (visual, auditory). Preliminary work suggests that olfactory cues may also be involved in human social communication. For example, past work suggests that biologically related individuals (e.g. mother and child) can accurately recognize each other based on olfactory cues alone [13,14,15,16]. Further, individuals may also be able to recognize (via olfactory cues) those who are *not* genetically related to them, such as romantic partners [17] or friends [18,19]. Thus, olfactory dysfunction could have interpersonal impacts beyond the familial domain and into the broader social network.

In line with this, recent work has linked olfactory dysfunction with reduced social network size [20], and suggests that this effect is particularly strong for women [21]. Interestingly, work involving individuals with congenital anosmia (i.e. those born without a sense of smell) has also shown that olfactory dysfunction may lead to different interpersonal deficits for men and women. For example, anosmic women may experience increased social insecurity, while anosmic men report fewer sexual partners [22]. Specifically, this finding might suggest that for women, the effects of olfactory dysfunction may be noticeable on a broader scale (e.g. within the broader social network), whereas for men, effects of dysfunction may be reserved to specific social behaviors (e.g. those related to sexual behavior). Thus, it appears that the social implications of olfactory dysfunction differ based on gender. Such findings may in part be due to noted differences in olfactory functioning, with women showing consistently better olfactory functioning relative to men [23]. In addition to women outperforming men on odor identification tests, women also appear to have superior olfactory sensitivity, meaning they are able to detect odors at lower concentrations relative to men [24].

Despite such evidence that olfactory dysfunction may adversely affect interpersonal functioning in humans, no research to date has directly examined whether these social deficits may mediate the link between olfaction and mortality. Further, in past work examining the link between social life and olfaction, social life is typically measured via a composite covering multiple dimensions of social relationships (e.g. composites made up of number of network members and frequency of socializing). Combining these separate constructs makes it difficult to determine what aspect of social life (the sheer number of network members or frequency of in-person contact) is driving the observed effect.

Thus, in the present work, we sought to determine whether social network size alone—as in the sheer number of network members—mediated the olfactory-mortality link in a sample of older adults. Given that this is a novel area of research in humans, we wanted to begin by examining older adults, where greater variability in olfactory function could be observed. Past work examining congenital anosmia (e.g. those born without a sense of smell) generally includes a wide age range (e.g. 18–50 years [22]). This work shows effects of olfactory dysfunction on adult relationships, possibly up until age 50; thus, there is reason to believe these effects will continue into old age. Further, there is a great deal of work highlighting the continued importance of social relationships (specifically for physical health) into older adulthood (e.g. [25]).

We focused on social network size, rather than focusing on other, more structural social network characteristics, as it served as a relatively objective and quantitative measure regarding social ties. We recognize that social network size alone does not capture every feature of complex social networks. For this reason, we then examined the specific underlying features of social networks, aside from size, that may explain this mediating effect, namely: physical closeness and emotional closeness. Individuals with reduced social network size may experience 1) reduced physical closeness, or less frequent in-person physical contact with other individuals, and 2) reduced emotional closeness, or reduced feelings of social support and increased feeling of loneliness/isolation. Physical closeness is especially interesting in the context of smell given that perception of olfactory cues requires proximity to other individuals. Emotional closeness, in contrast, has been consistently linked to physical health and mortality outcomes [5], but it is not constrained by proximity. For this reason, emotional closeness may play less of a role in terms of mediating the link between olfaction and mortality. Finally, given past work in patients with congenital anosmia, we examined whether gender moderated the hypothesized effects. Following past work in this area, we expected that associations between olfaction and social variables would be stronger for women.

## Materials and methods

Analyses were conducted using the National Social Life, Health, and Aging Project (NSHAP) dataset, from which the original olfactory-mortality link was reported. This longitudinal dataset is comprised of a sample of older adults (*N* = 3,005) and consists of two waves of data collection occurring five years apart (Wave 1: 2005–2006, Wave 2: 2010–2011). Data collection for Waves 1 and 2 of the NSHAP was approved by the Institutional Review Boards of The University of Chicago and NORC. All respondents provided written, informed consent. Participants with missing data on any key variables were excluded from analyses, leaving *N* = 2,264. It is important to note that the age profile of this restricted sample (*M*_age_ = 69.08, *SD* = 7.74, range: 57–85) closely resembles that of the full NSHAP dataset (*M*_age_ = 69.30, *SD* = 7.85, range: 57–85).

### Olfactory dysfunction

Olfactory dysfunction was measured using a validated field olfaction test [26] that was administered during Wave 1 of data collection. Participants were presented with five scented felt-tipped pens and were asked to identify the correct odor of each pen in a forced-choice, multiple choice format (four possible answers). The number of incorrect answers (ranging from 0 to 5) served as a measure of olfactory dysfunction, with higher scores indicating worse sense of smell. Refusals to provide an answer were coded as incorrect (as in [2]).

### Measures of social life

Social network size was measured by a composite score of two items: self-reported number of friends and number of close relatives. Following past work [21], the presence of a romantic partner was not explicitly included as a separate component of social network size. Based on the instructions for identifying number of friends and close relatives (see S1 Table), we assumed that individuals would have likely included their spouse/partner as a person they considered a close friend. Thus, we determined that including a separate variable for presence of a spouse/partner would have been redundant.

Physical closeness was assessed by a composite score of two measures: frequency of physical contact with others during the past year (five items, e.g. “In the last 12 months, how often have you greeted someone with an embrace, kiss, or pat on the back?”) and frequency of in-person socializing with others (one item). The five items measuring frequency of physical contact were averaged together to create a contact score.

Emotional closeness was assessed by a composite score of two measures: perceptions of social support (12 items, e.g. “How often can you open up to your [family/friends/partner] if you need to talk about your worries?”) and feelings of loneliness (three items, e.g. “How often do you feel that you lack companionship?” [27]). The 12 items measuring social support were averaged together and the three loneliness items were averaged together to create a social support score and a loneliness score.

Each composite was a sum of the two variables after z-transformation. (See S1 Table for details on measures.)

### Determination of mortality

Wave 1 participants were re-contacted at Wave 2 to determine mortality status (e.g. alive/deceased). For those that could not be contacted directly, researchers interviewed proxies when possible in order to establish mortality status. The status of 10 Wave I participants could not be determined, and thus their final disposition at Wave II was unknown. These participants were thus excluded from analyses.

## Results

We conducted moderated mediation analyses using a combination of ordinary least squares regression and binomial logistic regression with 95% quasi-Bayesian confidence intervals (CI) based on 10,000 Monte Carlo simulations using the mediation package (v. 4.4.6) [28] for R [29]. All models included demographic and health-related variables, following [2]. (See S2–S4 Tables for full models.)

### Social network size as a mediator

Similar to [21], greater olfactory dysfunction at Wave I was associated with smaller social network size for females (*β* = –.29, *p <* .001), but not for males (*β* = –.06, *p =* .197; *p*_interaction_ < .001). Smaller social networks were associated with higher mortality risk overall (*β* = –.13, *p =* .001). Moreover, network size was a partial mediator of the olfactory dysfunction-mortality link for females only (females: CI: [0.001, 0.006], *p* = .001; males: CI: [–0.0004, 0.002], *p* = .199; CI_difference_: [–0.006, –0.0001], *p*_difference_ = .039; S1 Fig). In order to examine the features of social network size that might help to explain its mediating role in the relationship between olfactory dysfunction and mortality, we next examined two underlying features of social networks—physical closeness and emotional closeness—as mediators.

### Physical closeness as a mediator

**Fig 1:**
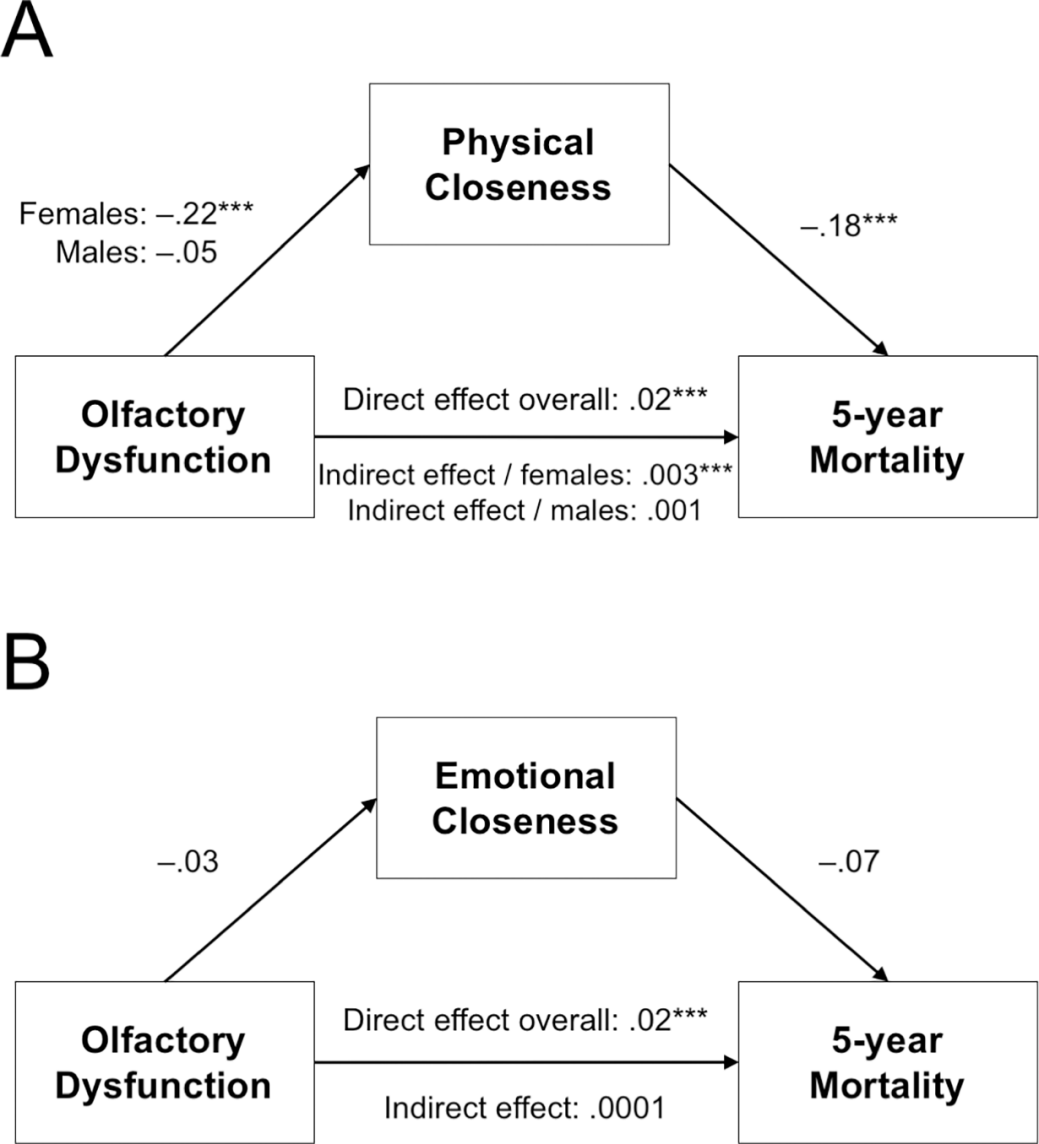
Physical closeness, but not emotional closeness, partially mediates the olfactory-mortality link for females. (A) Model summary for physical closeness as a mediator, with gender as a moderator on the *a* path. (B) Model summary for emotional closeness as a mediator. Asterisks indicate significant coefficients (*p < .05, **p < .01, ***p < .001). Unstandardized coefficients are reported.

Greater olfactory dysfunction was associated with decreased physical closeness for females (*β* = –.22, *p* < .001), but not for males (*β* = –.05, *p* > .250; *p*_interaction_ = .003). Decreased physical closeness was associated with higher mortality risk overall (*β* = –.18, *p* < .001). Interestingly, for females only, physical closeness partially mediated the olfactory dysfunction-mortality link (females: CI: [0.002, 0.006], *p* < .001; males: CI: [–0.001, 0.002], *p* > .250; CI_difference_: [–0.006, –0.00004], *p*_difference_ = .047; Fig 1A).

### Emotional closeness as a mediator

For emotional closeness, the gender X olfactory dysfunction interaction was not significant (*p* > .250), indicating that the effect of emotional closeness did not differ between males and females. Thus, standard mediation models were run with gender as a covariate.

Olfactory dysfunction was not associated with emotional closeness (*β* = –.03, *p* = .217), and emotional closeness was not associated with mortality risk (*β* = .07, *p* = .217). Emotional closeness did not mediate the olfactory-mortality link (CI: [–.0008, .0002], *p* > .250; Fig 1B).

### Comparing network size and physical closeness as mediators

Finally, we examined whether network size or physical closeness was more important in explaining the olfactory-mortality link for females. After controlling for physical closeness, network size was no longer a mediator for females (CI: [–.00003, .003]). In contrast, physical closeness remained a significant mediator for females, even after controlling for social network size (CI: [.0005, .003]).

## Discussion

We show, for the first time, that social network size, as measured by a composite representing only sheer size of network, partially mediated the olfactory-mortality link in females. Further inspection of this finding suggests that reduced in-person physical contact with others, rather than smaller social networks per se, may partially drive the link between olfactory dysfunction and physical health. In other words, the mediating effect of social network size was no longer significant after controlling for physical closeness, while the effect of physical closeness remained even after controlling for social network size. Feelings of emotional closeness were not associated with mortality status and emotional closeness did not serve the same mediating role as physical closeness in the olfaction-mortality link.

These findings fit with prior research showing that olfactory function is critical for motivating physical contact, particularly for females [9,21]. Moreover, we show, for the first time, that olfactory-induced physical contact, rather than social or emotional contact more generally, may be critical for the link between olfactory function and longevity. Due to the cross-sectional nature of the NSHAP data examined here (e.g. simultaneous measurement of olfactory dysfunction and social variables), we cannot say for certain that olfactory dysfunction causes reduced physical closeness. At the present time, this is unfortunately a consistent methodological limitation of most research in this area. Experimental work should be a goal of future work, as it is critical to understanding whether olfactory dysfunction can directly impact social behavior in humans.

Reward mechanisms may partially underlie the finding that olfaction dysfunction is associated with reduced physical closeness. Past work suggests that perceiving human body odors activates reward-related neural regions. For example, smelling the body odors of infants activates reward regions in new mothers [30]. This reward mechanism is hypothesized to be critical for mother-infant bonding and motivating maternal caregiving. Additional evidence suggests that this reward response to body odors may extend beyond kin: Women high in social openness show increased activation in reward regions in response to body odors of strangers [31]. In line with these findings, individuals with olfactory impairments qualitatively report that being unable to smell close others is upsetting or anxiety-promoting [32]. To the extent that the rewarding nature of body odors motivates individuals to seek out physical contact, olfactory dysfunction may interrupt the motivation to seek in-person social contact via disrupted reward processes.

Moreover, associations between body odors and reward may be stronger within females [33], which may partly account for the observed gender difference. Past work in this area has either only examined females (e.g. [30,31]), or has not been sufficiently powered to detect gender differences (e.g. [20]). Characteristics of the odor perceiver (e.g. gender, personality, relationship to target) may be especially important in understanding the differences in reward activation in response to human body odors. In addition, given that females tend to have superior olfactory functioning relative to males [23,24], the interpersonal impact of dysfunction may be more evident. This effect may be exaggerated in the present sample, given that sensory decline is common in older adults. Both males and females likely experienced decline in olfactory functioning due to aging. However, declines in functioning may be more noticeable and impactful for females, given that prior to decline, they presumably had superior functioning relative to men.

While a large body of research has directly tied social support and loneliness to health outcomes, less work has focused on how physical closeness could exert similar effects in humans. However, some research suggests that engaging in affective touch can have positive consequences, including lower self-reports of physical pain [34], reduced neural activation in threat-related regions [35], as well as a reduced heart rate and cortisol in response to a stressor [36]. In line with this, it has been hypothesized that interpersonal physical contact may serve to regulate individuals physiologically [37,38]. Thus, increased physical closeness may allow more opportunities to engage in this type of touch with other individuals in the social network, thus leading to better regulation of different biological systems (such as stress responding or immune system responses), and more favorable physical health outcomes as a result.

Finally, these findings may be particularly relevant today, as many of our social interactions occur through online interactions where no physical contact occurs. To the extent that olfactory-induced physical contact is critical for health and longevity, virtual social interactions may not fulfill this need. Virtual social interactions preclude both olfactory cue transmission and physical contact from occurring. Future work is needed to better understand how the absence of olfactory cues in these online interactions may serve to minimize physical health benefits that might otherwise be conferred by increased physical contact in these contexts.

## Supporting information

S1 Fig Model with social network size as mediator. (TIFF)

S1 Table Details of measures. (DOCX)

S2 Table Summary of model with social network size as mediator. (DOCX)

S3 Table Summary of model with physical closeness as mediator. (DOCX)

S4 Table Summary of model with emotional closeness as mediator. (DOCX)

S1 File Full syntax for analyses. (R)
