# Supplementary figures and images for: The role of social relationships in the link between olfactory dysfunction and mortality
Source: PLoS One. 2018 May 16;13(5):e0196708. doi: 10.1371/journal.pone.0196708 (PMC5955501; doi:10.1371/journal.pone.0196708)

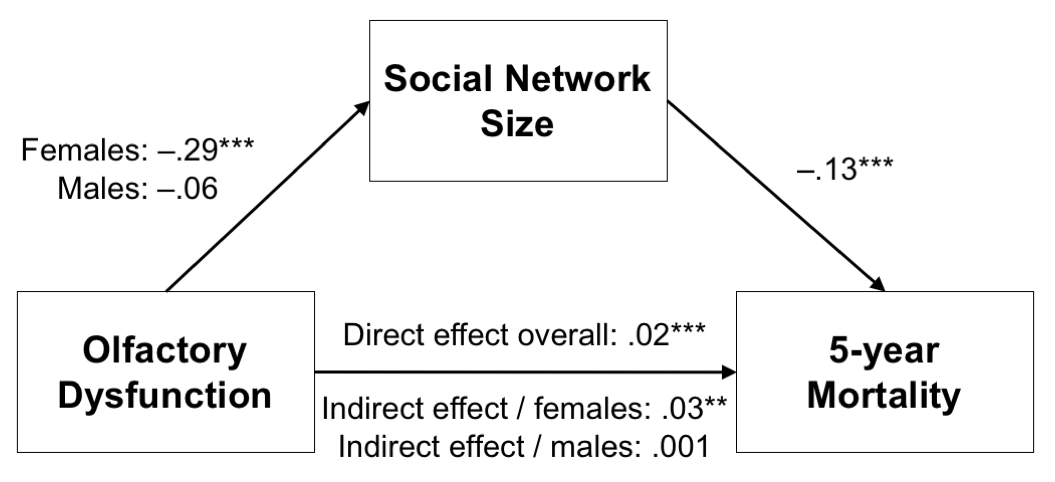

Supplement: S1 Fig — (TIFF) [file pone.0196708.s001.tiff]
