# Supplementary material for: The role of social relationships in the link between olfactory dysfunction and mortality
Source: PLoS One. 2018 May 16;13(5):e0196708. doi: 10.1371/journal.pone.0196708 (PMC5955501; doi:10.1371/journal.pone.0196708)
Supplement: S1 Table — (DOCX) [file pone.0196708.s002.docx]

**Table S1. Details of Measures.**

| Measure |  | Item |  | Response Choices |
| --- | --- | --- | --- | --- |
| **Social Network Size** |  |  |  | (0) None  (1) One  (2) 2-3  (3) 4-9  (4) 10-20  (5) More than 20 |
| *Number of Friends* |  |  |  |  |
| Think about the people you consider to be your friends, both your closest friends and people with whom you are pretty good friends. About how many friends would you say that you have? | | |  |  |
| *Number of Close Relatives* | | |  |  |
| Other than your partner, how many family members or relatives do you have whom you feel close to? | | |  |  |
| **Physical Closeness** |  |  |  |  |
| *Physical Contact* |  |  |  | (0) Never  (1) Less than once a year  (2) About once or twice a year  (3) Several times a years  (4) About once a month  (5) About once every week  (6) Several times a week |
| In the last 12 months, how often have you engaged in the following activities: | | |  |  |
| 1. Petting or touching a cat, dog, or other pet? | | |  |  |
| 1. Greeting someone with an embrace, kiss, or pat on the back? | | |  |  |
| 1. Hugging, kissing, caressing, or other close physical contact with your partner? | | |  |  |
| 1. Playing or cuddling with a grandchild or other child? | | |  |  |
| 1. Hugging, holding, or other close physical contact with another adult (other than your partner)? | | |  |  |
| *In-Person Socializing* | | |  |  |
| In the past 12 months, how often did you get together socially with friends or relatives? | | |  |  |

**Table S1. Details of Measures.** *(continued)*

| Measure | | |  | Item |  | Response Choices | | |  |
| --- | --- | --- | --- | --- | --- | --- | --- | --- | --- |
| **Emotional Closeness** |  |  | | | | |  |  | |
| *Social Support* |  |  | | | | |  | (0) Hardly ever (or never)  (1) Some of the time  (2) Often | |
| 1. How often can you open up to your partner if you need to talk about your worries? | | | | | | |  |  |  |
| 1. How often can you rely on your partner for help if you have a problem? | | | | | | |  |  |  |
| 1. How often does your partner make too many demands on you? | | | | | | |  |  |  |
| 1. How often does your partner criticize you? | | | | | | |  |  |  |
| 1. How often can you open up to family members if you need to talk about your worries? | | | | | | |  |  |  |
| 1. How often can you rely on family members for help if you have a problem? | | | | | | |  |  |  |
| 1. How often do family members make too many demands on you? | | | | | | |  |  |  |
| 1. How often do family members criticize you? | | | | | | |  |  |  |
| 1. How often can you open up to your friends if you need to talk about your worries? | | | | | | |  |  |  |
| 1. How often can you rely on your friends for help if you have a problem? | | | | | | |  |  |  |
| 1. How often do your friends make too many demands on you? | | | | | | |  |  |  |
| 1. How often do your friends criticize you? | | | | | | |  |  |  |
| *Loneliness* |  |  | | | | |  |  |  |
| 1. How often do you feel that you lack companionship? | | | | | | |  |  |  |
| 1. How often do you feel left out? | | | | | | |  |  |  |
| 1. How often do you feel isolated from others? | | | | | | |  |  |  |

**Table S1. Details of Measures.** *(continued)*

| Measure |  | Item |  | Correct Answer |
| --- | --- | --- | --- | --- |
| **Olfactory Function** |  |  |  |  |
| *I have five pens that contain a smell of something familiar. For each pen, identify the smell using the four answer choices.* | | |  |  |
| 1. (a) Chamomile   (b) Raspberry  (c) Rose  (d) Cherry | | |  | Rose |
| 1. (a) Smoke   (b) Glue  (c) Leather  (d) Grass | | |  | Leather |
| 1. (a) Orange   (b) Blueberry  (c) Strawberry  (d) Onion | | |  | Orange |
| 1. (a) Bread   (b) Fish  (c) Cheese  (d) Ham | | |  | Fish |
| 1. (a) Chive   (b) Peppermint  (c) Pine  (d) Onion | | |  | Peppermint |
