# Supplementary material for: The role of social relationships in the link between olfactory dysfunction and mortality
Source: PLoS One. 2018 May 16;13(5):e0196708. doi: 10.1371/journal.pone.0196708 (PMC5955501; doi:10.1371/journal.pone.0196708)
Supplement: S2 Table — (DOCX) [file pone.0196708.s003.docx]

**Table S2. Summary of Model with Social Network Size as Mediator.**

|  |  | Consequent | | | | | | | | |
| --- | --- | --- | --- | --- | --- | --- | --- | --- | --- | --- |
|  |  | Social Network Size | | |  | 5-Year Mortality | | | | |
|  |  | *R*^2^ = .04, *p* < .001 | | |  |  | | | | |
| Antecedent |  | Coeff. | *SE* | *p* |  | Coeff. | | *SE* | | *p* |
| Olfactory Dysfunction |  | –.06 | .04 | .197 |  | .23 | .06 | | < .001 | |
| Social Network Size |  | — | — | — |  | –.13 | .04 | | < .001 | |
| Gender* |  | .25 | .09 | .005 |  | –.23 | .14 | | .105 | |
| Olfactory Dysfunction X Gender |  | –.23 | .06 | < .001 |  | — | — | | — | |
| Age |  | .01 | .005 | .005 |  | .06 | .01 | | < .001 | |
| African American (vs. white) |  | –.24 | .10 | .018 |  | .14 | .19 | | .457 | |
| Hispanic (vs. white) |  | –.51 | .12 | < .001 |  | –.25 | .25 | | .320 | |
| Other (vs. white) |  | .35 | .22 | .108 |  | .04 | .45 | | .925 | |
| Education^†^ |  | .04 | .03 | .119 |  | –.17 | .06 | | .004 | |
| Heart Attack |  | .04 | .12 | .721 |  | .10 | .21 | | .635 | |
| Heart Failure |  | .07 | .14 | .611 |  | 1.12 | .21 | | < .001 | |
| Stroke |  | .11 | .13 | .363 |  | .53 | .20 | | .009 | |
| Diabetes |  | .02 | .09 | .833 |  | .44 | .16 | | .006 | |
| Hypertension |  | –.05 | .07 | .497 |  | –.14 | .14 | | .327 | |
| COPD/Emphysema |  | –.11 | .11 | .313 |  | .24 | .20 | | .210 | |
| Liver Damage |  | .80 | .35 | .024 |  | 1.62 | .50 | | .001 | |
| Cancer^‡^ |  | .17 | .11 | .107 |  | .45 | .19 | | .015 | |
| Constant |  | –.88 | .33 | .008 |  | –6.66 | .69 | | < .001 | |

Only participants with complete data on all variables were included in the analyses (*N* = 2,264).

* Coded as 0 for males, 1 for females. ^†^Highest degree earned; treated as continuous.  ^‡^Excluding skin cancer.
