# Supplementary material for: The role of social relationships in the link between olfactory dysfunction and mortality
Source: PLoS One. 2018 May 16;13(5):e0196708. doi: 10.1371/journal.pone.0196708 (PMC5955501; doi:10.1371/journal.pone.0196708)
Supplement: S3 Table — (DOCX) [file pone.0196708.s004.docx]

**Table S3. Summary of Model with Physical Closeness as Mediator.**

|  |  | Consequent | | | | | | | | |
| --- | --- | --- | --- | --- | --- | --- | --- | --- | --- | --- |
|  |  | Physical Closeness | | |  | 5-Year Mortality | | | | |
|  |  | *R*^2^ = .09, *p* < .001 | | |  |  | | | | |
| Antecedent |  | Coeff. | *SE* | *p* |  | Coeff. | | *SE* | | *p* |
| Olfactory Dysfunction |  | –.05 | .04 | .267 |  | .24 | .06 | | < .001 | |
| Physical Closeness |  | — | — | — |  | –.18 | .04 | | < .001 | |
| Gender* |  | .37 | .08 | < .001 |  | –.19 | .14 | | .166 | |
| Olfactory Dysfunction X Gender |  | –.17 | .06 | .003 |  | — | — | | — | |
| Age |  | –.02 | .004 | < .001 |  | .06 | .01 | | < .001 | |
| African American (vs. white) |  | –.93 | .10 | < .001 |  | –.01 | .20 | | .973 | |
| Hispanic (vs. white) |  | –.29 | .11 | .013 |  | –.22 | .25 | | .378 | |
| Other (vs. white) |  | –.30 | .21 | .140 |  | –.002 | .44 | | .996 | |
| Education^†^ |  | .10 | .02 | < .001 |  | –.15 | .06 | | .009 | |
| Heart Attack |  | .12 | .12 | .314 |  | .11 | .21 | | .615 | |
| Heart Failure |  | –.04 | .13 | .767 |  | 1.11 | .21 | | < .001 | |
| Stroke |  | .01 | .12 | .935 |  | .52 | .20 | | .011 | |
| Diabetes |  | –.05 | .08 | .514 |  | .42 | .16 | | .009 | |
| Hypertension |  | –.03 | .07 | .650 |  | –.12 | .14 | | .398 | |
| COPD/Emphysema |  | .02 | .10 | .821 |  | .27 | .20 | | .165 | |
| Liver Damage |  | .47 | .34 | .160 |  | 1.59 | .50 | | .001 | |
| Cancer^‡^ |  | –.02 | .10 | .859 |  | .43 | .19 | | .021 | |
| Constant |  | 1.36 | .31 | < .001 |  | –6.32 | .69 | | < .001 | |

Only participants with complete data on all variables were included in the analyses (*N* = 2,264).

* Coded as 0 for males, 1 for females. ^†^Highest degree earned; treated as continuous.  ^‡^Excluding skin cancer.
