# Supplementary material for: The role of social relationships in the link between olfactory dysfunction and mortality
Source: PLoS One. 2018 May 16;13(5):e0196708. doi: 10.1371/journal.pone.0196708 (PMC5955501; doi:10.1371/journal.pone.0196708)
Supplement: S4 Table — (DOCX) [file pone.0196708.s005.docx]

**Table S4. Summary of Model with Emotional Closeness as Mediator.**

|  |  | Consequent | | | | | | | | |
| --- | --- | --- | --- | --- | --- | --- | --- | --- | --- | --- |
|  |  | Emotional Closeness | | |  | 5-Year Mortality | | | | |
|  |  | *R*^2^ = .07, *p* < .001 | | |  |  | | | | |
| Antecedent |  | Coeff. | *SE* | *p* |  | Coeff. | | *SE* | | *p* |
| Olfactory Dysfunction |  | –.03 | .02 | .217 |  | .26 | .06 | | < .001 | |
| Emotional Closeness |  | — | — | — |  | .07 | .06 | | .217 | |
| Gender* |  | .51 | .05 | < .001 |  | –.25 | .14 | | .074 | |
| Age |  | –.01 | .003 | .004 |  | .06 | .009 | | < .001 | |
| African American (vs. white) |  | .14 | .07 | .054 |  | .18 | .19 | | .350 | |
| Hispanic (vs. white) |  | –.13 | .09 | .139 |  | –.19 | .25 | | .442 | |
| Other (vs. white) |  | .18 | .16 | .254 |  | –.01 | .45 | | .991 | |
| Education^†^ |  | –.03 | .02 | .075 |  | –.17 | .06 | | .004 | |
| Heart Attack |  | –.01 | .09 | .884 |  | .09 | .21 | | .666 | |
| Heart Failure |  | .25 | .10 | .011 |  | 1.08 | .21 | | < .001 | |
| Stroke |  | .12 | .09 | .187 |  | .51 | .20 | | .011 | |
| Diabetes |  | –.09 | .06 | .176 |  | .44 | .16 | | .005 | |
| Hypertension |  | .09 | .05 | .067 |  | –.15 | .14 | | .302 | |
| COPD/Emphysema |  | .16 | .08 | .046 |  | .24 | .20 | | .214 | |
| Liver Damage |  | .51 | .25 | .044 |  | 1.45 | .50 | | .003 | |
| Cancer^‡^ |  | .17 | .08 | .027 |  | .41 | .19 | | .027 | |
| Constant |  | .37 | .24 | .119 |  | –6.63 | .69 | | < .001 | |

Only participants with complete data on all variables were included in the analyses (*N* = 2,264).

* Coded as 0 for males, 1 for females. ^†^Highest degree earned; treated as continuous.  ^‡^Excluding skin cancer.
